# Inflammatory pseudotumor mimicking primary hepatic malignant tumor with hepatitis B virus-related cirrhosis: A case report

**DOI:** 10.3892/ol.2013.1386

**Published:** 2013-06-10

**Authors:** QINGHONG KE, LE FAN, XIN DUAN, ZENGLEI HE, SHUSEN ZHENG

**Affiliations:** Department of Hepatobiliary and Pancreatic Surgery, The First Affiliated Hospital, College of Medicine, Key Laboratory of Combined Multiorgan Transplantation, Ministry of Health, Zhejiang University, Hangzhou, Zhejiang 310003, P.R. China

**Keywords:** inflammatory pseudotumor, liver, hepatitis B virus, malignant tumor

## Abstract

Inflammatory pseudotumors (IPT) of the liver are fairly uncommon lesions. IPTs are difficult to diagnose due to the absence of specific symptoms. The correct diagnosis is easily missed, particularly in livers with hepatitis B virus (HBV)-related cirrhosis. The current study presents the case of a 58-year-old male with a ten-year history of HBV infection, who was diagnosed with a primary liver tumor by computed tomography (CT) and magnetic resonance imaging (MRI). The α-fetoprotein levels ranged within normal limits. A local resection was performed and the histopathological analysis identified IPT of the liver. The patient recovered well following surgery.

## Introduction

Inflammatory pseudotumors (IPTs) arise most commonly from the lungs (1). Histologically, IPTs are characterized by proliferating fibrovascular tissue with an infiltration of inflammatory cells, including plasma cells, lymphocytes and eosinophils (2). IPTs of the liver are rare and are often mistaken as malignant tumors. Computed tomography (CT) scans and magnetic resonance imaging (MRI) are of certain value when forming a differential diagnosis. However, in specific cases, particularly those with hepatitis B virus (HBV)-related cirrhosis, it is extremely difficult to establish a definite diagnosis by radiological imaging. For these cases, a correct differential diagnosis of IPT from the malignant tumor is of great importance to prevent the delay of necessary treatment (3).

The current study presents the case of liver IPT with HBV-related cirrhosis, which was misdiagnosed as a primary hepatic malignant tumor. The final diagnosis of IPT was made by a post-operative pathological examination. Written informd consent was obtained from the patient.

## Case report

### Clinical presentation

A 58-year-old male was referred to the First Affiliated Hospital (Hangzhou, China) following detection of a lesion in the right lobe of the liver by ultrasonography of the abdomen. Upon admission, the patient was free from symptoms and in good general health, without jaundice. The liver and spleen were not palpated, since the patient had no hepatomegaly and only had mild splenomegaly, and there was no sign of any abdominal mass. In addition, the patient had a 10-year history of HBV infection.

### Pathological analysis

**Figure 1.**
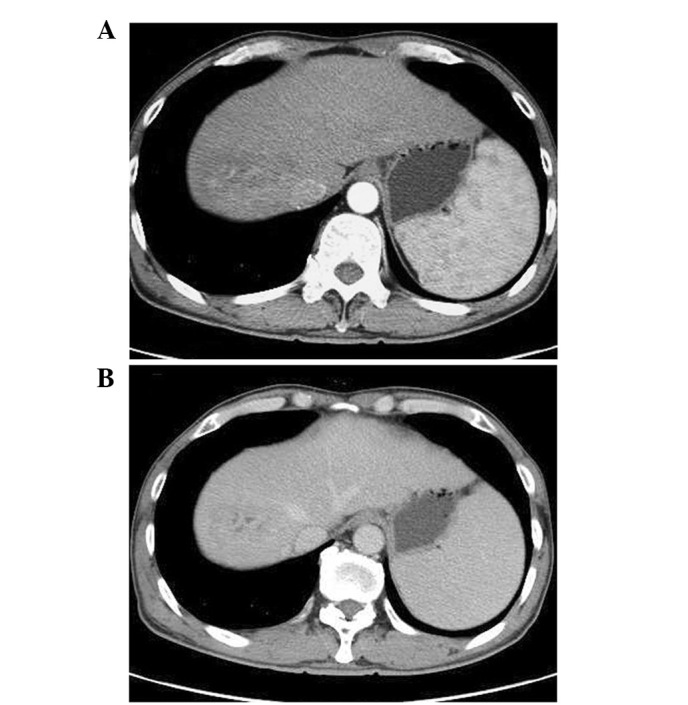
Contrast computed tomography scan demonstrated that the lesion featured a (A) mild enrichment on the arterial phase and (B) hypointense on the venous phase.

**Figure 2.**
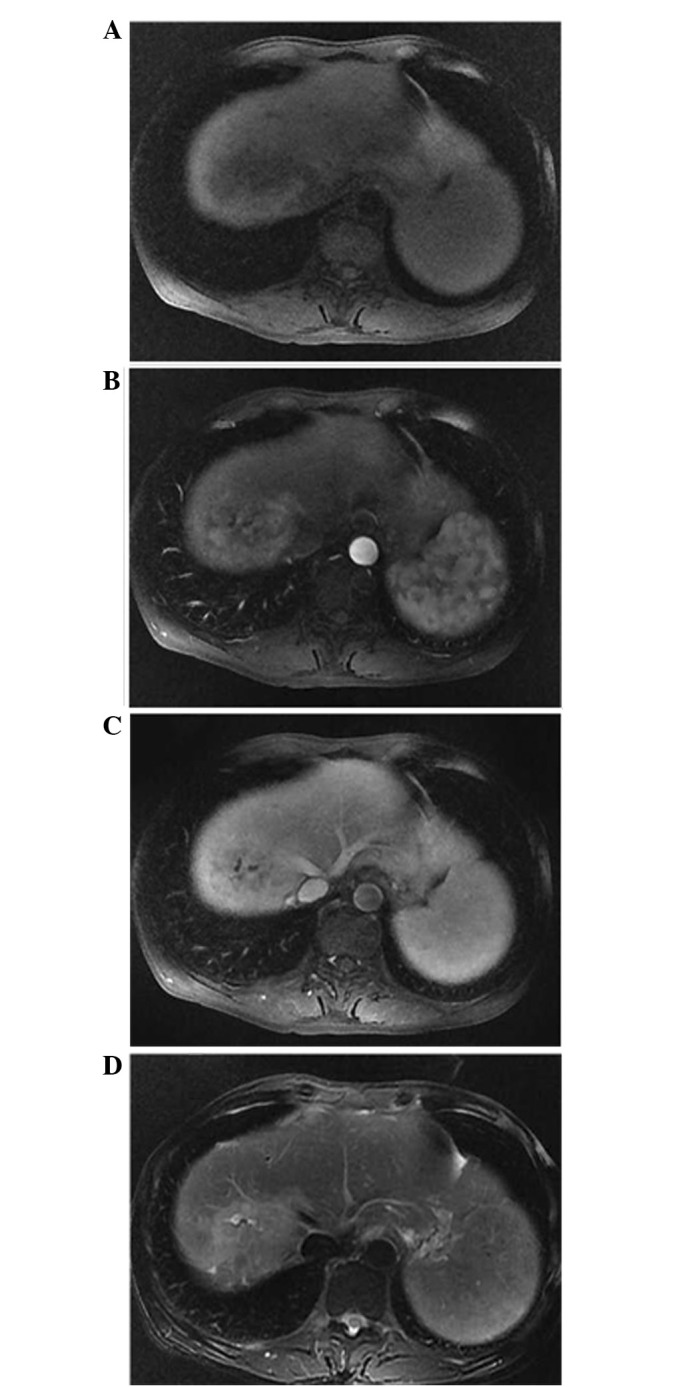
Magnetic resonance imaging (MRI) demonstrating that the inflammatory pseudotumor was (A) hypointense on the unenhanced T1-weighted image, (B) partially hyperintense on the enhanced T1-weighted image, (C) hypointense on the hepatobiliary phase image (T1-weighted) and (D) partially hyperintense on the unenhanced T2-weighted image.

Laboratory investigations revealed normal liver function test results. The hepatitis serology for HBsAg was positive and no hepatitis C infection was identified. In addition, no leukocytosis was observed and normal AFP, CA19-9 and CEA levels were detected. The upper GI endoscopy and colonoscopy results were normal. The abdominal CT (Fig. 1) and MRI (Fig. 2) examinations revealed a well-defined heterogeneous mass situated in Couinaud segment 8 and measuring 3.8×5.0 cm. The lesion featured a mild enrichment from the arterial phase in the CT and MRI, consistent with a malignancy. The initial diagnosis was of a primary hepatic malignant tumor. During surgery, mild liver cirrhosis was identified. The tumor was located in Couinaud segment 8 and had clear boundaries. A local resection was performed and the intra-operative blood loss was measured at 300 ml. The patient recovered well following the surgery and was consequently discharged on the ninth post-operative day.

### Histological analysis

**Figure 3.**
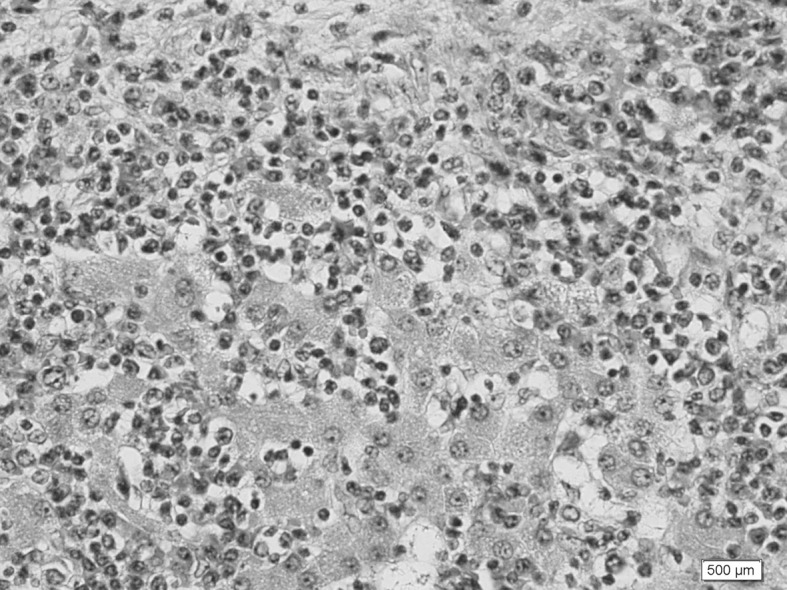
Histopathological analysis showing fibrosis and numerous infiltrating lymphocytes, largely plasma plasma cells. HE staining (magnification, ×400).

Macroscopically, the cut surface of the resected specimen was that of a yellowish-white tumor, which was 4.0 cm in diameter. A microscopic examination revealed a process with benign characteristics, which included numerous infiltrating lymphocytes, mainly plasma cells (Fig. 3). These histological observations confirmed the final diagnosis of a hepatic IPT.

## Discussion

Liver IPT was first described in 1953 by Pack and Baker (2). To date, the etiology and pathogenesis of IPTs remain unknown. Liver IPTs are associated with a number of diseases, including Crohn’s disease, diabetes mellitus, Sjögren’s syndrome, gout, chronic cholangitis, primary sclerosing cholangitis, Kostmann’s disease and autoimmune pancreatitis (3). The majority of patients usually present with a fever and abdominal pain (3), and a small number of patients suffer from jaundice caused by idiopathic inflammatory structures of the extrahepatic biliary tree. Clinical manifestations and imaging are similar to those of a tumor with the exception of the benign biological behavior and the properties of spontaneous regression following treatment with antibiotics (4) or non-steroidal anti-inflammatory drugs (5). CT scans and MRI are the main methods to establish the diagnosis. A CT scan usually reveals lesions with variable contrast enhancement. IPTs may present with a hypovascular character in the CT scan and manifest as a low signal intensity (hypointense) on T1-weighted images with moderate to high signal intensities (hyperintense) on T2 sequences in MRI. The imaging appearance of an IPT is diverse and depends on the proportion and distribution of inflammatory cells and fibrosis within the lesion (5). Generally, tumor markers are not useful, as the levels of the majority of markers fall within the normal range. In specific cases, a diagnosis is extremely difficult to make.

For cases of suspected IPT, the importance of percutaneous needle biopsy has been emphasized, and due to the risk of spontaneous regression, unnecessary surgery must be avoided (6). In the current case, a percutaneous needle biopsy was not performed. The imaging appearance of the IPT indicated a malignant character, consistent with the patient history of HBV-related cirrhosis. The lesion was located on the surface of the Couinaud segment. As we were concerned over the relatively high rate of hemorrhaging following a possible percutaneous needle biopsy, as well as the risk of needle tract seeding, a surgical resection without needle biopsy was performed. Needle tract seeding has been reported to occur in 5.1% of patients with hepatocellular carcinoma who have undergone percutaneous needle biopsy (7). Although the case was ultimately proved to be that of an IPT by a post-operative pathological examination, in our opinion, a needle biopsy should not be utilized as a routine diagnostic tool if a lesion is strongly suspected to be of malignant character. Active surgical resection must be the first choice. While hepatectomy is dangerous in patients with poor health, a liver biopsy must be considered in these cases to avoid unnecessary surgical procedures.

In general, IPTs are considered to represent benign lesions, however, the correct treatment protocol for these pseudotumors remains controversial. Certain studies have reported that lesions are likely to be completely resolved following treatment with antibiotics. However, specific lesions have recurred following this treatment protocol. By contrast, IPTs have never been reported to recur following surgical resection. We recommend that short-term observation should be performed in patients diagnosed with IPT. In addition, for cases where the lesion is difficult to differentiate from the malignancy or is associated with high risk factors, including HBV-related cirrhosis, surgical resection must be considered.
